# Satisfaction With Outdoor Activities Over Time Among Long-Term Services and Supports Recipients

**DOI:** 10.1093/geroni/igaa057.614

**Published:** 2020-12-16

**Authors:** Justine Sefcik, Karen Hirschman, Darina Petrovsky, Liming Huang, Nancy Hodgson, Liza Behrens, Mary Naylor

**Affiliations:** 1 Drexel University, Philadelphia, Pennsylvania, United States; 2 NewCourtland Center for Transitions and Health, Philadelphia, Pennsylvania, United States; 3 University of Pennsylvania, Philadelphia, Pennsylvania, United States; 4 University of Pennsylvania, Cherry Hill, New Jersey, United States; 5 University of Pennsylvania School of Nursing, Philadelphia, Pennsylvania, United States

## Abstract

Approximately 85% of older adults have at least one chronic health condition. The onset of chronic health conditions and mobility issues can constrain activities, including outdoor recreation. There is limited knowledge of older adults receiving long-term services and supports (LTSS) and their satisfaction with outdoor activities over time after enrolling in services. This study examined predictors of change in ratings of satisfaction with outdoor activities. A secondary analysis was conducted of data involving structured interviews with older adults (N=470) over the first two years of receiving LTSS (Health-Related Quality of Life: Elders in Long-Term Care; R01AG025524). Participants lived in assisted living communities, nursing homes, or their home. A single item on satisfaction with outdoor activities (assessed using a 5-point Likert scale: not at all to extremely satisfied) was the primary outcome. Mixed effects linear regression modeling using a backward elimination process was used for building a final multivariable model. In the final model, older age (p<0.001) and higher overall quality of life ratings (p<0.001) at baseline were associated with slower rates of increase in outdoor satisfaction over time. Higher education level (p=0.035) at baseline was associated with a faster rate of increase in outdoor satisfaction over time. Additionally, those who moved into an assisted living community (p=0.024) or nursing home (p=0.016) at baseline were associated with faster rates of increase in outdoor satisfaction over time compared to those in the home. Knowledge of factors influencing satisfaction with outdoor activities can assist interdisciplinary teams implement interventions for individual or organizational changes.

